# Liang-Ge Decoction Ameliorates Coagulation Dysfunction in Cecal Ligation and Puncture-Induced Sepsis Model Rats through Inhibiting PAD4-Dependent Neutrophil Extracellular Trap Formation

**DOI:** 10.1155/2023/5042953

**Published:** 2023-04-29

**Authors:** Wenju He, Qiang Xi, Huantian Cui, Pingping Zhang, Rui Huang, Taihuan Wang, Dongqiang Wang

**Affiliations:** ^1^Department of Integration of Traditional Chinese and Western Medicine, First Central Hospital Affiliated to Nankai University, Tianjin First Central Hospital, Tianjin, China; ^2^Department of Practice and Education, Tianjin University of Traditional Chinese Medicine, Tianjin, China; ^3^Shandong Provincial Key Laboratory of Animal Cell and Developmental Biology, School of Life Sciences, Shandong University, Qingdao, China; ^4^Department of Graduate School, Tianjin University of Traditional Chinese Medicine, Tianjin, China

## Abstract

Liang-Ge (LG) decoction could ameliorate coagulation dysfunction in septic model rats. However, the mechanism of LG in treating sepsis still needs to be clarified. Our current study established a septic rat model to evaluate the effect of LG on coagulation dysfunction in septic rats first. Second, we investigated the effect of LG on NET formation in septic rats. Finally, NETs and PAD4 inhibitors were further used to clarify if LG could improve the mechanism of sepsis coagulation dysfunction by inhibiting NET formation. Our findings indicated that treatment with LG improved the survival rate, reduced inflammatory factor levels, enhanced hepatic and renal function, and reduced pathological changes in rats with sepsis. LG could also alleviate coagulation dysfunction in septic model rats. Besides, LG treatment reduced NETs formation and decreased PAD4 expression in neutrophiles. In addition, LG treatment showed a similar result in comparison to the treatment with either NET inhibitors or PAD4 inhibitors alone. In conclusion, this study confirmed that LG has therapeutic effects on septic rats. Furthermore, the improvement of coagulation dysfunction in septic rats by LG was achieved through inhibiting PAD4-mediated NET formation.

## 1. Introduction

Sepsis is a common complication for critically ill patients [1] and a medical condition in which the body's response to an infection leads to organ dysfunction and can be life-threatening if not treated promptly [2]. The incidence and case fatality rate of sepsis remains high worldwide, where the rate of sepsis among patients in hospitals in the United States is approximately 6%, and the case fatality rate is 15% [3]. In contrast, the rate of sepsis among patients in hospitals in China is 8.68%, and the case mortality rate of severe sepsis is 33.5%–48.7% [4]. The high costs caused by sepsis impose a heavy economic burden on social and medical care [5], and a World Health Organization resolution published in *N Engl J Med* in 2017 identified sepsis as a top global health problem [6].

Western medicine treats sepsis mostly with anti-infectives, fluid resuscitation, immunomodulation, symptomatic, and supportive therapies. Anti-infective therapy, as a basic treatment, has been demonstrated with severe antibiotic resistance problems recently [7, 8]. In contrast, the widely accepted early goal-directed therapy has been shown in several large-scale randomized controlled studies that it cannot reduce the long-term case mortality rate in patients with sepsis [9]. Furthermore, related studies of novel therapies, such as using immune cells, inflammatory mediators, and the coagulation system as targets, have also failed to lower the mortality rate in sepsis [10]. To date, there is a lack of drug that has been definitively shown to be effective in treating sepsis, which remains a major global health problem. Several studies have demonstrated that traditional Chinese medicine (TCM) is effective in improving clinical symptoms and the survival rate of sepsis [11]. Furthermore, a comprehensive examination of previous studies, known as a meta-analysis, has discovered that the administration of the Xue-Bi-Jing injection in conjunction with ulinastatin significantly decreases the mortality rate among septic patients, curbs the occurrence of multiorgan failure, and reduces the concentration of serum proinflammatory factors in affected individuals [12]. Additionally, animal experiments have shown that 6-gingerol improves liver damage in septic rats by activating the NRF2 signaling pathway [13]. Another TCM, Xi-Jiao-Di-Huang decoction, lowers the mortality rate of septic rats and decreases the expression levels of NF*κ*Bp65 and HIF-1*α* [14]. Xuan-Bai-Cheng-Qi decoction inhibits inflammatory response of septic rats by regulating gut microbiota [15]. Therefore, understanding the underlying mechanism of how TCM works in treating sepsis is essential for advancing the modern application of TCM.

The neutrophil extracellular trap (NET) is a new mechanism for the neutrophil extracellular killing of pathogens, a recently discovered technique. NETs are a complex network of fibers that are produced by neutrophils when they release their nuclear components into the extracellular space. This phenomenon can be triggered by various pathogens or drugs that stimulate the activation of neutrophils [16]. NETs have various roles, including limiting the spread of pathogens and secreting bactericidal proteins; furthermore, neutrophils die after NETs are released, a process called NETosis [17]. During sepsis, activated neutrophils use NETs to capture and kill pathogens. However, abnormally increased NETs damage endothelial cells, activate platelets, and deliver tissue factors, promoting thrombosis, leading to coagulation disorders, and exacerbating organ dysfunction in sepsis [18]. Further studies have shown that tissue factors (TFs) released from NETs in sepsis induce thrombosis and cause coagulation dysfunction by affecting PAR-1 [19]. Coagulation dysfunction in sepsis can be alleviated by inhibiting NET formation.

Liang-Ge decoction (LG), consisting of *Lophatherum gracile* Brongn., *Mirabilite*, *Glycyrrhiza glabra* L., *Gardenia jasminoides* J.Ellis, *Mentha canadensis* L., *Scutellaria baicalensis* Georgi, *Forsythia suspensa* (Thunb.) Vahl, and *Rheum palmatum* L., has potential as a treatment for the early stages of sepsis. Our previous study also showed that LG significantly reduced the mortality rate and improved coagulation dysfunction in septic rats [20]. However, the exact way in which LG works to treat sepsis needs to be explained. The purpose of this study was to evaluate the effects of LG on coagulation dysfunction and NET formation in septic rats. To do so, the septic rat model was established in this study through the use of the cecal ligation and puncture (CLP) procedure, which is widely recognized as an appropriate method to induce sepsis in laboratory animals. Finally, NET inhibitors were further used to investigate whether LG could improve the mechanism of sepsis coagulation dysfunction by inhibiting NET formation. Our research aimed to provide insight into the therapeutic potential of LG in addressing these aspects of sepsis, with the goal of contributing to the development of new and effective treatments for this condition.

## 2. Materials and Methods

### 2.1. Preparation of LG

First, we weighed LG (20 g of *Forsythia suspensa* (Thunb.) Vahl, 10 g of *Mirabilite*, 6 g of *Glycyrrhiza glabra* L., 10 g of *Gardenia jasminoides* J.Ellis, 6 g of *Mentha canadensis* L., 10 g of *Scutellaria baicalensis* Georgi, 6 g of *Rheum palmatum* L., and 10 g of *Lophatherum gracile* Brongn.). Then, we added eight times the volume of water and decocted for 30 min, which was finally concentrated to 3 g crude drug/mL.

### 2.2. Animal and Reagents

Detailed information and cat numbers are included in supplementary materials (available here).

### 2.3. Animal Studies

There were 360 male SD rats used in this study. All rats were housed in a quiet environment with *ad libitum* feeding. This experiment was approved by the Ethics Committee of Tianjin First Central Hospital (approval no. 2021-SYDWLL-000266).

### 2.4. Establishment of a Septic Rat Model

The septic rat model was established by the classic CLP method [21]. The rats were fasted for 12 hours and then anesthetized using isoflurane gas. An incision was made on the midabdomen, and then the cecum was isolated and ligated 5 mm from the ileocecal region using surgical suture. After ligating, the cecum was perforated twice on both sides of the intestinal wall by a syringe needle (20 G) 0.8–1 cm far from the distal appendiceal root of the ligature. The content in intestine was squeezed out a little and a drainage strip was inserted into the perforation hole. The incision in the abdominal cavity was sutured after the cecum was placed back in its original position.

### 2.5. Experimental Grouping and the Drug Administration Protocol

**Figure 1:**
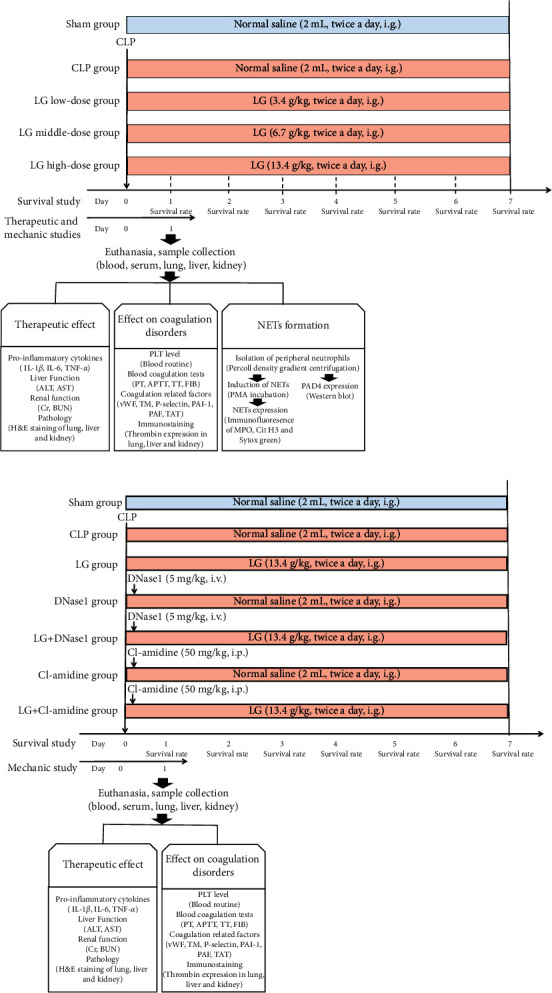
Experimental design for the study of therapeutic effects of LG on sepsis (a) and study of NET and PAD4 inhibitors (b).

There are 150 rats divided into five groups (Sham, CLP, LG low-dose (LLG), LG middle-dose (MLG), and LG high-dose (HLG)) randomly in a study to evaluate the therapeutic effects, coagulation function, and formation of NETs in septic rats. The sepsis model was established in rats from the CLP, LLG, MLG, and HLG groups using the CLP method, while the rats in the Sham group only underwent an incision and suture of the abdominal wall without undergoing the CLP procedure. Following the establishment of the septic rat model, rats in the LLG, MLG, and HLG groups were gavaged at doses of 3.9 g crude LG/kg, 7.8 g crude LG/kg, and 15.6 g crude LG/kg, respectively, while rats in the Sham and CLP groups were gavaged with the vehicle every 12 h. The LG dose was determined by applying a conversion formula to adapt the dosage for experimental animals, in which the medium dose was the human equivalent dose with the low dose being half of the medium dose and the high dose being twice the medium dose (Figure 1(a)).

Furthermore, in the study using NETs and peptidyl arginine deiminase 4 (PAD4) inhibitors, a total of 210 rats were divided into 7 groups (Sham, CLP, LG, DNase1, LG + DNase1 (LGDN), Cl-amidine, and LG + Cl-amidine (LGCl)) groups randomly. Rats in the Sham group only underwent an incision and suture of the abdominal wall without undergoing the CLP procedure, while other groups used CLP to establish the septic model. After modeling, the Sham and CLP groups were gavaged with the vehicle. The LG group was gavaged with 15.6 g crude LG/kg; the DNase1 group was given a single tail vein injection of DNase1 5 mg/kg 1 h after modeling, followed by gavage of 2 mL saline; the LGDN group was given a single tail vein injection of DNase1 5 mg/kg 1 h after modeling, followed by gavage of 15.6 g crude LG/kg; the Cl-amidine group was intraperitoneal injected with Cl-amidine 50 mg/kg 1 h after modeling, followed by gavage of 2 mL saline; and the LGCl group was intraperitoneal injected with Cl-amidine 50 mg/kg 1 h after modeling, followed by gavage of 15.6 g crude LG/kg. The gavage frequency was every 12 h for rats in all groups (Figure 1(b)).

For survival statistics, 15 rats were randomly selected from each group and the survival rate was monitored daily for a period of 7 days following the administration of CLP. The survival curve was then plotted based on the data collected. The remaining rats in each group were sacrificed 24 hours after drug administration to establish the septic model. Blood, lung, liver, and kidney samples were collected from these rats for further testing.

### 2.6. Platelet, Coagulation, and Serum Biochemical Tests

Blood samples were collected from the abdominal aorta. The collected blood (200 *μ*L) was placed in an ethylenediaminetetraacetic acid (EDTA)-K2 anticoagulated tube and mixed gently. The platelet count (PLT) was performed using a fully automated hemocytometer. An additional 200 *μ*L of blood was collected and placed in a sodium citrate anticoagulated tube and mixed gently. Subsequently, the levels of prothrombin time (PT), activated partial thromboplastin time (APTT), thrombin time (TT), and fibrinogen (FIB) were measured using an automated blood coagulation analyzer. The platelet count (PLT) and coagulation tests were carried out within 2 hours of blood collection. Furthermore, the serum was collected and the levels of creatinine (Cr), blood urea nitrogen (BUN), alanine aminotransaminase (ALT), and aspartate aminotransferase (AST) were tested using an automatic biochemical analyzer.

### 2.7. H&E Staining

The tissues collected from the lung, liver, and kidney of each group were prepared for examination by undergoing a process of fixation and paraffin embedding. Afterward, 3-micrometer sections of the tissue were cut and stained with H&E, followed by washing and mounting. These sections were then meticulously examined under a light microscope to detect the histopathological changes.

### 2.8. ELISA

The serum was obtained by centrifuging the collected blood at room temperature (10 min, 3000 rpm). The serum levels of various inflammation and coagulation markers were measured. All protocols were performed according to the manufacturer's protocol.

### 2.9. Immunohistochemically Staining

The collected lung, liver, and kidney were subjected to a multistep process to visualize and quantify the expression of thrombin in the tissue. The tissue was first fixed and paraffin embedded and then cut into 3- micrometer sections. Following dewaxing and hydration, antigen retrieval was performed. Then, sections were permeabilized and blocked. Next, the sections were incubated with primary antibody (1 : 100) overnight at 4°C, followed by washing. After that, the secondary antibody (1 : 10000) was added and incubated for 1 h at room temperature and washed. The sections were then treated with conventional DAB for color development and restained, dehydrated, and made transparent. After mounting, the area of positive expression in tissue was observed. Additionally, the extent of positive expression was determined by measuring the average optical density (AOD) using Image-J software.

### 2.10. Neutrophil Extraction

A 5 mL of collected blood samples from each group were placed in an EDTA-K2 anticoagulation vacuum blood collection tube. The blood sample was then added to the peripheral blood neutrophil isolation kit to isolate neutrophils from the rats of each group based on the instructions. Detailed protocols are included in supplementary material.

### 2.11. NET Induction and Immunofluorescence Detection of NET Levels

Purified neutrophils were cultured, resuspended, and added to 12-well plates with a concentration of 8 × 10^5^ cells per well. The cells were then incubated for 1 hour. A poly-D-lysine-coated 12-well chamber slide was added to each well. The medium containing 89% DMEM, 10% FBS, and 1% penicillin-streptomycin was used. Then, cells were incubated with phorbol 12-myristate 13-acetate (PMA; 100 nM) for 3 h to induce the NET formation [22]. The cell slides were fixed, permeabilized, and blocked. Then, the slides were incubated with rabbit polyclonal to histone H3 (1 : 200) and MPO mouse monoclonal (1 : 1000) fluorescent antibodies at 4°C overnight. Secondary antibodies were added, and then it was incubated at room temperature for 1 h after washing. SYTOX Green nucleic acid dye (50 nM) was added and reacted at room temperature. After washing, sections were restained with DAPI, then washed again, mounted with an antifade agent, and the image was observed by immunofluorescence microscopy. The quantification of fluorescence intensity and area was performed using Image Pro Plus 6.0. The average optical density (AOD) was calculated as the integrated optical density (IOD) divided by the total area.

### 2.12. Western Blotting

The extracted peripheral blood neutrophils were lysed on ice and the supernatant was collected after centrifuging at 4°C (12,000 rpm, 10 min). The protein concentration of the obtained supernatant was tested using the BCA kit. After normalization of density, 20 *μ*g of each sample was obtained for SDS-PAGE at a density of 10%. The target proteins separated from the gel were transferred onto polyvinylidene fluoride (PVDF) membranes that had been excited by methanol. Next, the membranes were blocked in 5% skim milk, after which primary antibodies were added for incubation at 4°C overnight. After washing the membranes, secondary antibodies were added and the samples were incubated at room temperature for 2 hours. The protein expression levels were then detected through enhanced chemiluminescence and the resulting bands were analyzed using Image-J software. Detailed information about the antibody is included in supplementary material.

### 2.13. Statistical Processing

The experimental results were analyzed using the SPSS, and the data were expressed as $\bar{x}$ ± *S*. The *T*-test and one-way ANOVA were used to compare the means between multiple groups. A *p* value of less than 0.05 was considered statistically significant.

## 3. Results

### 3.1. Effects of LG on Survival Rate, Inflammatory Factor Levels, Hepatic and Renal Function, and Histopathological Changes in Septic Rats

**Figure 2:**
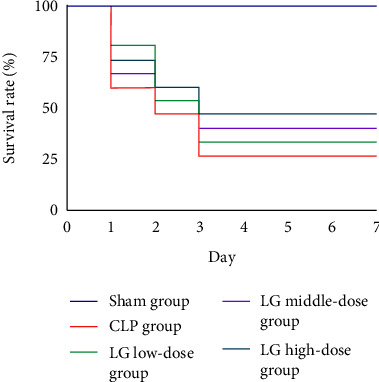
LG treatment induced the survival rate in septic rats. The Sham, CLP, LLG, MLG and HLG groups (*n* = 15 per group).

After 7 d of drug administration for modeling, the survival rate of the Sham, CLP, LLG, MLG, and HLG groups was 100%, 26.67%, 33.34%, 40%, and 46.67%, respectively (Figure 2).

**Figure 3:**
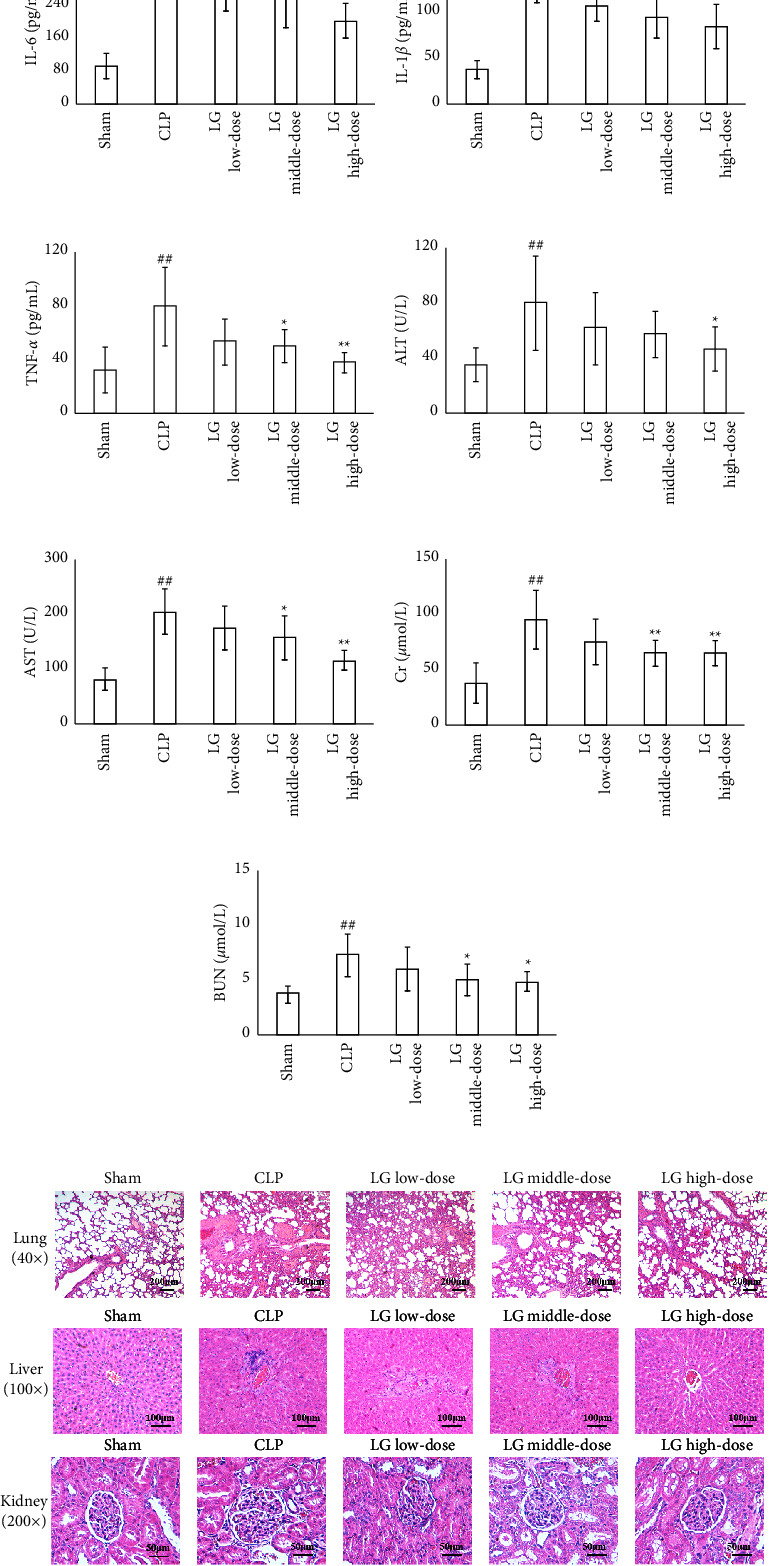
LG treatment ameliorated inflammatory factor levels, improved the hepatic and renal function, and reduced pathological changes in septic rats. (a–c) LG treatment decreased serum levels of cytokines in septic rats; (d and e) LG treatment decreased the activities of ALT (d) and AST (e) in serum; (f and g) LG treatment decreased the levels of Cr (f) and BUN (g) in serum; (h) H&E staining indicated that LG treatment reduced the pathological changes of the lung, liver, and kidney in septic model rats. The Sham, CLP, LLG, MLG, and HLG (*n* = 15, 8, 8, 10, and 11, respectively).

After 24 hours of treatment, the serum levels of cytokines were higher in the CLP group compared to the Sham group. In comparison to the CLP group, the serum level of IL-6 was reduced in the HLG group, and the serum levels of IL-1*β* were reduced in the LLG, MLG, and HLG groups. The serum level of TNF-*α* was also reduced in the MLG and HLG groups (Figures 3(a)–3(c)).

Serum liver function indicators showed that the activities of ALT and AST in serum were higher in rats of the CLP group than those of the Sham group. Compared with the CLP group, serum activities of ALT were reduced in HLG group rats; the serum activity of AST was reduced in rats from the MLG and HLG groups (Figures 3(d) and 3(e)). Furthermore, results of the renal function tests revealed that levels of Cr and BUN were notably higher in rats in the CLP group compared to those in the Sham group. The MLG and HLG interventions reduced serum levels of Cr and BUN (Figures 3(f) and 3(g)).

H&E staining results showed that LG could reduce the damage caused by CLP. The results from the lung testing revealed that alveolar wall thickening, telangiectasia, pulmonary mesenchymal hyperemia, and inflammatory cell infiltration were found in the CLP group. While after taking treatment of LG, the inflammatory cells in each LG-treated rat were reduced, the alveolar structure tended to be intact, and the pathological damage was significantly reduced dose-dependently. The staining results of liver showed hepatocytes in the CLP group were scattered, hepatic cords were disorganized, hepatocytes showed necrosis, and inflammatory cell infiltration was observed compared with the Sham group. Additionally, hepatocytes of rats in each LG dose group were arranged neatly, and the hepatocyte necrosis and the infiltration in the liver were alleviated. Furthermore, the results of kidney testing revealed that rats in the CLP group had focal lesion and atrophy of renal tubules, enlargement of the tubular lumen, and infiltration of renal mesenchymal inflammatory cells compared with the Sham group. Compared with the CLP group, the lesions were reduced in all LG-treated rats (Figure 3(h)).

### 3.2. Effect of LG on the Coagulation Function of Septic Rats

**Figure 4:**
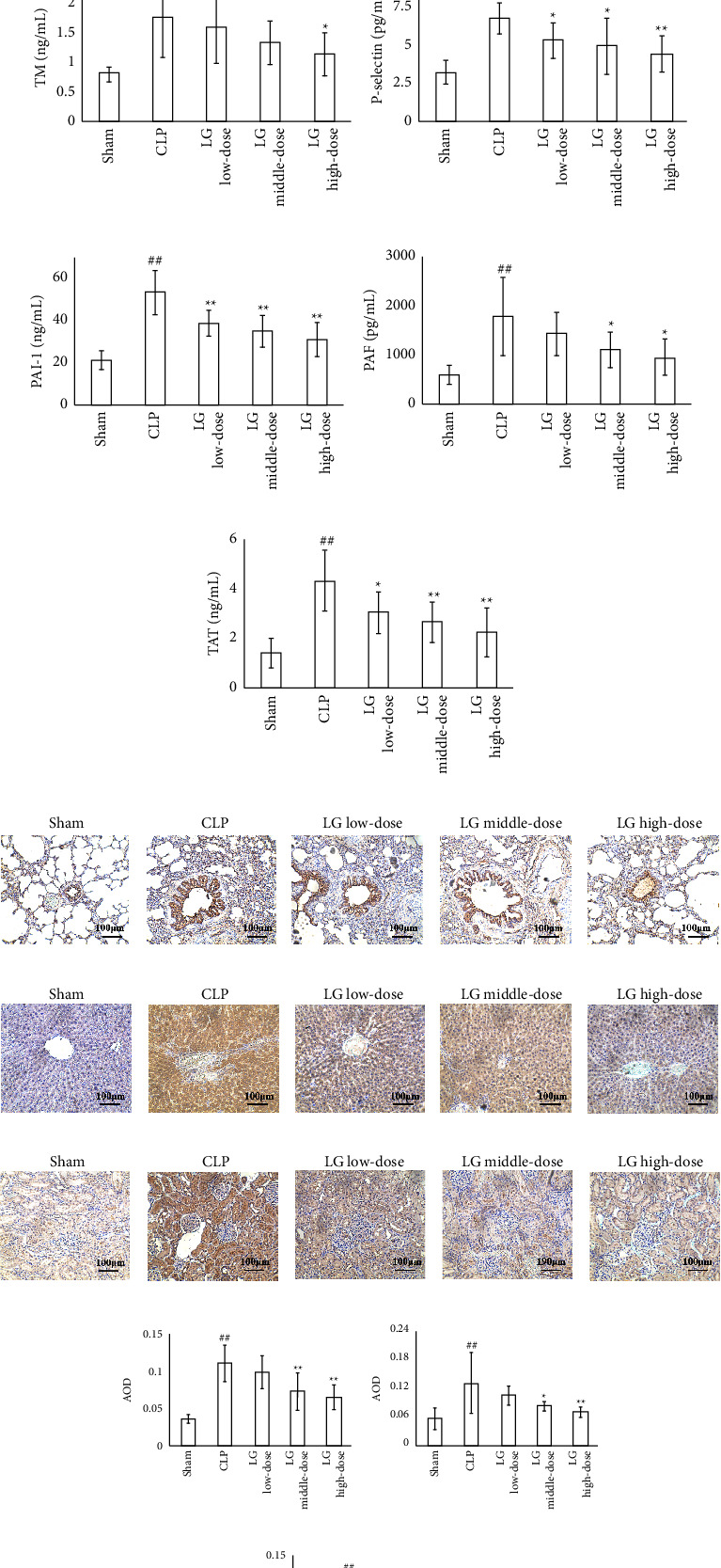
LG treatment ameliorated coagulation disorders in septic rats. (a) Blood routine showed that LG treatment increased the blood PLT count in septic rats; (b–g) ELISA tests showed that LG treatment decreased the levels of vWF (b), TM (c), P-selectin (d), PAI-1 (e), PAF (f), and TAT (g) in serum; (h–m) immunohistochemical staining showed that LG treatment decreased expression of thrombin in the lung (h and k), liver (i and l), and kidney (j and m) (100x). The Sham, CLP, LLG, MLG, and HLG groups (*n* = 15, 8, 8, 10, and 11, respectively).

The PLT level in peripheral blood was decreased in the CLP group compared with those of the Sham group and increased in rats of the MLG and HLG groups compared with that of the CLP group (Figure 4(a)). The findings from coagulation tests indicated that the APTT and PT values were extended and the levels of FIB were reduced in the CLP group compared to the Sham group. In contrast, APTT and PT values were shortened and the levels of FIB were increased in the MLG and HLG groups when compared to those of the CLP group (Table 1).

Furthermore, ELISA results showed that levels of coagulation factors were increased in serum of rats from CLP compared with those of the Sham group. The vWF level was decreased in all the LG-treated groups compared with the CLP group. Additionally, the TM level was decreased in the HLG group; the P-selectin level was decreased in all the LG-treated groups. The PAI-1 level was decreased in all the LG-treated groups; the PAF level was decreased in the MLG and HLG groups. Finally, the TAT level was decreased in all the LG-treated groups (Figures 4(b)–4(g)).

Immunohistochemical results revealed that the positive area of thrombin in the lung, liver, and kidney was increased in rats in the CLP group compared with those of the Sham group and the changes were reduced in the MLG and HLG groups (Figures 4(h)–4(m)).

### 3.3. Effects of LG on NET Formation and Expression of Neutrophil PAD4 in Septic Rats

**Figure 5:**
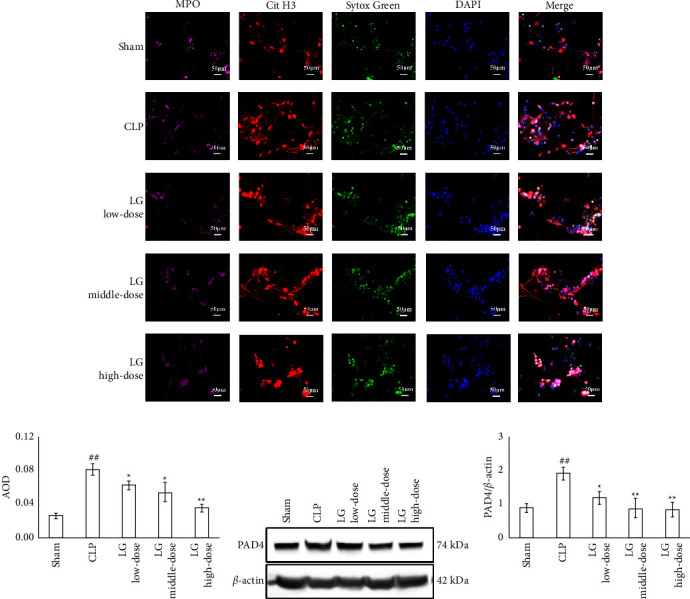
LG treatment inhibited the NET formation and decreased the PAD4 expression in neutrophiles in septic rats. (a and b) Neutrophiles were isolated from rat blood and treated with PMA to induce NET formation. Immunofluorescence showed that LG treatment decreased the expression of Cit H3 and SYTOX Green in peripheral neutrophiles (400x). (c and d) Neutrophiles were isolated from rat blood. Western blotting showed that LG treatment decreased the PAD4 expression in peripheral neutrophiles. The Sham, CLP, LLG, MLG, and HLG groups (*n* = 3 per group).

Immunofluorescence results showed that all cells had lobulated nuclei and expressed MPO. Additionally, Cit H3^+^Sytox Green^+^ fluorescence intensity was increased in rats of the CLP group compared with those of the Sham group, while it decreased in LLG, MLG, and HLG groups (Figures 5(a) and 5(b)). Western blotting results showed that expression of PAD4 was significantly increased in neutrophils of the CLP group compared with those of the Sham group. The PAD4 expression in neutrophils in HLG decreased compared to the CLP group (Figures 5(c) and 5(d)). Conclusively, the HLG group had the most significant effect in inducing the survival rate, improving the multiorgan function, coagulation dysfunction, and inhibiting NET formation in septic rats. Thus, the dosage of HLG was selected to be used in the following tests.

### 3.4. Effects of Inhibiting NET Formation and PAD4 on Survival, Inflammatory Factor Levels, Liver and Kidney Function, and Histopathological Changes in Rats with Sepsis Treated with LG

**Figure 6:**
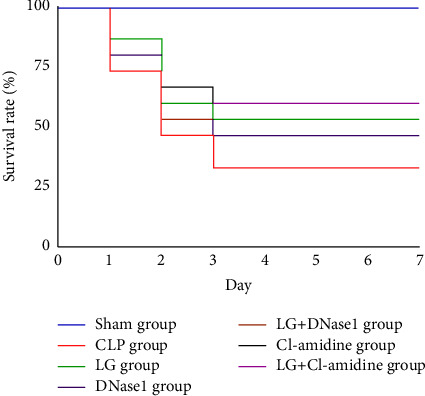
Effects of inhibiting NET formation and PAD4 on survival, in septic rats after LG treatment. The Sham, CLP, LG, DNase1, LGDN, Cl-amidine, and LGCl groups (*n* = 15 per group).

The intervention effect of LG in septic rats was investigated after inhibiting NET formation and PAD4 using inhibitor of NET or PAD4, respectively. After 7 d of LG intervention, the survival rate in the Sham and CLP groups was 100% and 33.33%, respectively. LG, DNase1, LGDN, Cl-amidine, and LGCl significantly increased the survival rate of septic rats, which was 53.33% in the LG group, 46.67% in the DNase1 group, 53.33% in the LGDN group, 53.33% in the Cl-amidine group, and 60% in the LGCl group (Figure 6).

**Figure 7:**
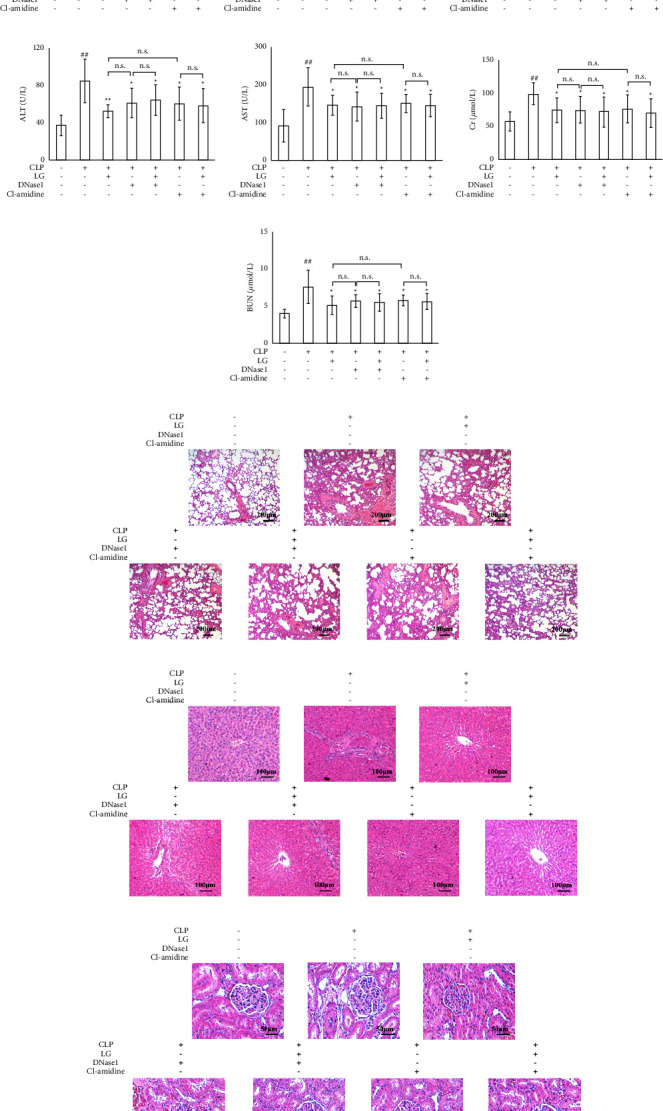
Effects of inhibiting NET formation and PAD4 on inflammatory factor levels, hepatic and renal function, and pathological changes in septic rats after LG treatment. (a–c) Effects of LG treatment on the serum levels of proinflammatory cytokines in septic rats after treatment with DNase1 and Cl-amidine; (d and e) effects of LG treatment on the serum activities of ALT (d) and AST (e) in septic rats after treatment with DNase1 and Cl-amidine; (f and g) effects of LG treatment on the serum levels of Cr (f) and BUN (g) in septic rats after treated with DNase1 and Cl-amidine; (h–j) effects of LG treatment on pathological changes in the lung (h, 40x), liver (i, 100x), and kidney (j, 200x) in septic rats after treated with the NET inhibitor (DNase1) and PAD4 inhibitor (Cl-amidine).The Sham, CLP, LG, DNase1, LGDN, Cl-amidine, and LGCl groups (*n* = 15, 7, 10, 9, 10, 10, and 9, respectively). n.s.: not statistically significant.

The results demonstrated that compared to the Sham group, levels of IL-6, IL-1*β*, and TNF-*α* were significantly increased in the serum of rats in the CLP group after administration for modeling. However, administration of LG, DNase1, LGDN, Cl-amidine, and LGCl showed a significant reduction in these markers of inflammation compared to the CLP group (Figures 7(a)–7(c)).

The findings of the serum liver function test indicated that the activity levels of ALT and AST were elevated in the CLP group in comparison to the Sham group. In contrast, the CLP group showed a substantial decrease in activity levels of ALT and AST when treated with LG, DNase1, LGDN, Cl-amidine, and LGCl (Figures 7(d) and 7(e)). Furthermore, results of the kidney function test revealed that the levels of Cr and BUN in the CLP group were notably higher than those in the Sham group. However, treatment with LG, DNase1, LGDN, Cl-amidine, and LGCl resulted in substantial reduced levels of Cr and BUN compared to the CLP group (Figures 7(f)–7(g)).

The H&E staining of lung tissues in the CLP group showed that the alveolar wall was thickened; there was telangiectasia, increased hyperemia of the pulmonary mesenchyme, and infiltration of inflammatory cells, compared to the Sham group. Furthermore, compared with the CLP group, inflammatory cells in the LG, DNase1, LGDN, Cl-amidine, and LGCl groups were reduced; the alveolar structure tended to be intact and pathological damages were significantly reduced (Figure 7(h)). The staining result indicated that the liver of rats in the CLP group showed evidence of cellular disorganization, necrosis, and inflammation compared to the liver of rats in the Sham group. Additionally, hepatocytes in the LG, DNase1, LGDN, Cl-amidine, and LGCl groups were neatly arranged, and the hepatocyte necrosis and inflammatory cell infiltration in the liver were reduced (Figure 7(i)). The results of the H&E staining of the kidneys revealed that when compared to the Sham group, rats in the CLP group displayed focal lesions and atrophy of the renal tubules, an enlargement of the tubular lumen, and infiltration of inflammatory cells in the renal mesenchyme. Furthermore, compared with the CLP group, renal lesions in the LG, DNase1, LGDN, Cl-amidine, and LGCl groups were significantly improved (Figure 7(j)). Importantly, there was no difference in inflammatory factor levels, the liver and kidney function, and histopathological changes between the LG and DNase1, LG and Cl-amidine, DNase1 and LGDN, and Cl-amidine and LGCl groups.

### 3.5. Effect of LG on the Coagulation Function in Septic Rats after Inhibiting NET Formation and PAD4

**Figure 8:**
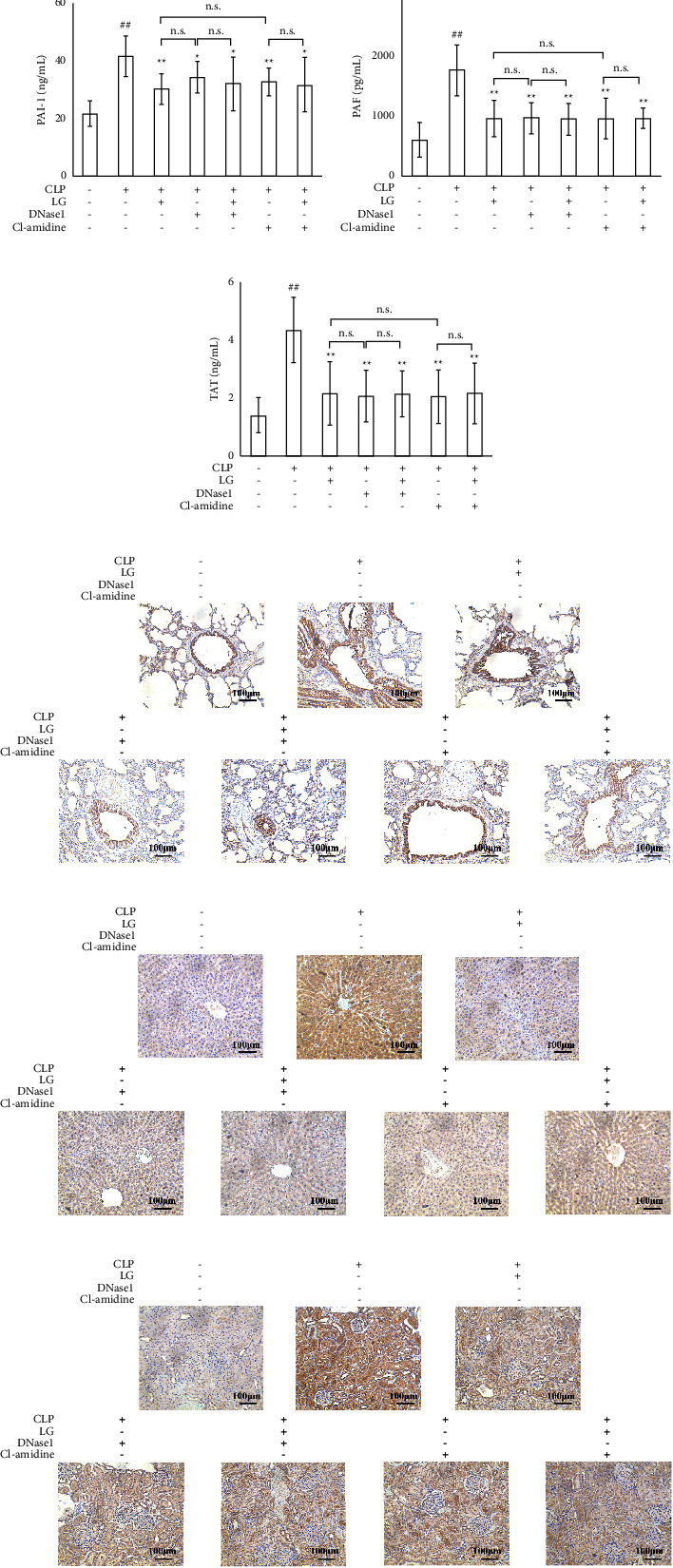
Effects of inhibiting NET formation and PAD4 on coagulation disorders in septic rats after LG treatment. (a) Effects of LG treatment on blood PLT count in septic rats after treated with DNase1 and Cl-amidine; (b–g) effects of LG treatment on the levels of vWF (b), TM (c), P-selectin (d), PAI-1 (e), PAF (f), and TAT (g) in septic rats after treated with DNase1 and Cl-amidine; (h–m) effects of LG treatment on the expression of thrombin on the lung (h and k), liver (i and l), and kidney (j and m) (100x) in septic rats after treated with DNase1 and Cl-amidine. The Sham, CLP, LG, DNase1, LGDN, Cl-amidine, and LGCl groups (*n* = 15, 7, 10, 9, 10, 10, and 9, respectively). n.s.: not statistically significant.

The results showed that the CLP group had a decrease in the peripheral blood PLT level compared to the Sham group, while treatment with LG, DNase1, LGDN, Cl-amidine, and LGCl resulted in an increase in the peripheral blood PLT level compared to the CLP group. This indicates that the various treatments were effective in restoring PLT levels, which play a crucial role in the process of hemostasis and blood clotting (Figure 8(a)). Furthermore, the results of the coagulation tests revealed that septic rats had prolonged APTT and PT values compared to those in the Sham group and also had significantly lower levels of FIB. Conversely, compared to the CLP group, rats in the LG, DNase1, LGDN, Cl-amidine, and LGCl groups had shorter APTT and PT values and higher FIB levels. These findings indicate the beneficial effects of treatment on restoring the coagulation function (Table 2).

Further examination of coagulation factors using ELISA revealed that when compared to the Sham group, the CLP group had elevated levels of coagulation factors. However, treatment with LG, DNase1, LGDN, Cl-amidine, and LGCl resulted in a noticeable reduction in these coagulation factors when compared to the CLP group (Figures 8(b)–8(g)).

The results of the immunohistochemical analysis revealed that the area of thrombin expression was found to be elevated in the lung, liver, and kidney of rats in the CLP group compared to those in the Sham group, as indicated by the presence of brown staining in the tissue sections. Furthermore, when compared to the CLP group, the expression of thrombin in the lung, liver, and kidney was found to be reduced in the LG, DNase1, LGDN, Cl-amidine, and LGCl groups, suggesting a potential protective effect of these treatments against thrombin-induced tissue damage (Figures 8(h)–8(m)). Additionally, there was no difference in coagulation related tests, and thrombin expression in lung, liver and kidney between LG and DNase1 groups, LG and Cl-amidine groups, DNase1 and LGDN groups, and Cl-amidine and LGCl groups.

## 4. Discussion

Here, we used the CLP method to establish a rat model with sepsis. CLP is a commonly used method for sepsis modeling that can simulate the pathological process of sepsis [21]. The results revealed that the survival rate of septic rats ranged from 20% to 40%, which was consistent with the results of other articles [23], and LG significantly increased the survival rate of septic rats, suggesting its therapeutic effect. The excessive activation of inflammatory response is important in early stages of sepsis [24]. The initial proinflammatory response to infection and injury is protective. However, excessive cytokine production is involved in multiple-organ damage in the development of sepsis [25]. Studies have shown that the levels of these factors are significantly elevated in rats from 6 hours to 48 hours after undergoing CLP [26, 27]. Likewise, our results revealed that levels of cytokines in serum were increased in the CLP group of rats 24 h after modeling. Also, the intervention of LG reduced the level of peripheral proinflammatory factors in septic rats. Liver and kidney functions are impaired in sepsis [28], and our results showed that levels of liver-and-kidney-functions-related biochemical indicators were significantly elevated in serum of septic model rats, while LG improved liver and kidney functions in septic rats.

Similarly, pathological staining also revealed that treatment with LG not only reduced the pathological changes in the liver and kidney tissues of septic rats, such as reducing inflammatory cell infiltration, alleviating apoptosis, and mitigating cellular damage, but also effectively ameliorated lung injury, which is a critical pathological change that occurs during sepsis. These results support the notion that LG has the potential to be beneficial in treating the pathological changes associated with sepsis. Lung injury is also a common and serious complication of sepsis [29]. Our results showed that after 24 h of CLP, many inflammatory cells infiltrated and hyperemia in the mesenchymal lung tissue of model rats, accompanied with alveolar and bronchial injury. LG significantly improved lung tissue damage in septic rats. Thus, the above results showed that LG has significant therapeutic effects on sepsis.

Numerous clinical and experimental studies have demonstrated the presence of varying degrees of coagulation dysfunction in sepsis [30–33]. Sepsis inflammation leads to vascular endothelial cell damage, secreting many procoagulant substances involved in the cascade reaction of coagulation or vasoconstriction, and causing coagulation dysfunction [34]. Dysfunction of the coagulation system in patients with sepsis includes increased procoagulant activity, decreased anticoagulant activity, and inhibited the fibrinolytic system. Furthermore, overactivation of the coagulation system and impaired anticoagulation mechanisms can further develop into disseminated intravascular coagulation (DIC), leading to organ dysfunction or death [35, 36]. The findings of the blood examination and coagulation analysis indicated that levels of PLT and FIB were diminished and duration of APTT and PT was prolonged in the septic rats. PLTs are small, disk-shaped cells that play a crucial role in blood clotting. They are formed by the fragmentation of megakaryocytes, which are found in the bone marrow. The cytoplasm of megakaryocytes is shed to form platelets, which circulate in the bloodstream and can aggregate at the site of an injury to help stop bleeding [37]. The decrease in PLT associated with sepsis is due to the host autoimmune response that triggers platelet activation, and the activated PLT interacts with activated endothelial cells and aggregates locally, decreasing the amount of PLT in peripheral blood [38]. FIB is a glycoprotein formed by the liver that can produce peptides under the action of thrombin, forming insoluble fibrin to exert hemostasis [39]. After the onset of sepsis, there would be excessive coagulation activation in the human body, where TFs induce thrombin production. The activated PLTs promote the conversion of FIB into fibrin, causing a large consumption of FIB [40]. Therefore, the FIB level in severe sepsis decreases significantly, and the value of FIB is negatively correlated with sepsis severity [41–43]. PT is an important indicator of the exogenous coagulation system, and PT prolongation is mostly seen in thrombotic diseases [44]. Furthermore, APTT is the most reliable indicator to assess the level of endogenous coagulation factors, and a prolonged APTT often indicates a higher risk of thrombosis in the body [45]. Our study found that LG treatment has a positive impact on coagulation dysfunction in septic rats. Specifically, LG administration was observed to increase PLT and FIB levels while also reducing the time of APTT and PT. These results suggest that LG has the ability to regulate the coagulation function in sepsis. Additionally, we examined effects of LG on various coagulation-associated markers. The results suggested that LG intervention could reduce serum levels of all 6 factors in septic rats, further demonstrating its potential in regulating the coagulation function in sepsis. Disruption of vascular endothelial cells occurs after the onset of sepsis, allowing inflammatory cells and fluid to infiltrate into the tissue mesenchyme while inducing a procoagulant state, leading to tissue microthrombus and ischemia [46]. Markers of endothelial cell damage, such as vWF and TM, are elevated in sepsis [47]. Furthermore, P-selectin, a membrane glycoprotein with a relative molecular mass of 140 KDa is mainly present on the *α*-granule membrane within PLTs and is a crucial marker of PLT activation. The P-selectin level in the serum of patients with sepsis is significantly elevated, causing excessive PLT activation [48, 49]. The PAI-1 is a crucial inhibitor of the activation of plasminogen *in vivo*. It is a single-strand glycoprotein and serine protease inhibitor, mainly synthesized by vascular endothelial cells. There, it binds to the plasminogen activator and rapidly inactivates to exert antifibrinolytic effects, reducing fibrin degradation and causing fibrin aggregation [50]. In sepsis, cytokines, such as IL-l*β*, TNF-*α*, bacterial endotoxin, and lipopolysaccharide, can increase PAI-1 synthesis and secretion in endothelial cells, inhibiting the fibrinolytic system [51–53]. PAF can promote PLT aggregation and induce thrombosis, and its elevated levels in the serum of patients with sepsis can lead to the abnormal circulatory function in the tissues and organs, causing damage to the body [54]. Studies have also found that increased PAF levels in patients with sepsis lead to pathological changes in the structure of endothelial cells, increasing their permeability, causing tissue edema and capillary leakage, and aggravating the systemic inflammatory response of the body [55, 56]. TAT is widely used as a sensitive marker of coagulation system activation, as its levels directly reflect the production of thrombin, which is a key component in the coagulation process. Furthermore, both coagulation and anticoagulation systems are activated during DIC onset, resulting in a significant increase in plasma TAT levels [57]. Similarly, immunohistochemical results showed that the intervention of LG reduced the expression of thrombin in the lung, liver, and kidney, further confirming that LG improves levels of coagulation-related factors in peripheral blood of septic rats and inhibits expression of thrombin in tissues and organs, thus improving the systemic coagulation dysfunction in septic rats.

NET overactivation is a crucial factor causing coagulation dysfunction in sepsis [58, 59]. We further investigated the effect of LG on the NET level in septic rats. First, we purified peripheral blood neutrophils from rats and used PMA to induce NET production, followed by immunofluorescence staining of the cells. PMA is a commonly used inducer of NETs, and studies have shown that a 100 nM intervention for 3 h significantly induces NET production in neutrophils [22]. MPO is a specialized enzyme found in the azurophilic granules of neutrophils and is widely used as a marker for identifying and quantifying the presence of neutrophils in a sample. This makes MPO a valuable tool for studying the neutrophil function and activation in various physiological and pathological processes, including inflammation and infection [60]. The results suggest that all of the cells in the study were positive for MPO. The use of H3 Cit with SYTOX Green staining has been widely recognized as an effective method for NET detection, as it allows for the specific identification of NETs through the visualization of DNA fibers in combination with a nucleic acid stain. This approach has been widely used in various studies to assess the formation and presence of NETs in septic conditions [61]. The results suggested that the level of NETs in neutrophils was elevated in septic rats, and LG inhibited production of NET. Additionally, we examined effect of LG on PAD4 expression in neutrophils of septic rats. This process of converting of arginine to citrulline by peptidylarginine deiminases (PADs) is a modification that occurs after protein synthesis. This modification can greatly impact the function, stability, and location of the protein. The role of PADs has been implicated in various diseases, such as autoimmune disorders and cancer, as their role in physiological and pathological processes is an ongoing area of research [62]. PADs have five isoforms, and PAD4 can mediate the citrullination of the arginine residues of the nucleus histones, thereby dedensifying chromatin and promoting NET formation [63]. PAD4-deficient neutrophils cannot release NETs under stimulation [64]. The results revealed that the intervention of LG reduced the expression of PAD4 in neutrophils, implying that it might improve the coagulation dysfunction in sepsis by reducing the expression of PAD4 in neutrophils and, in turn, inhibiting the formation of NETs in septic rats.

To further demonstrate the mechanism of action of LG in improving coagulation dysfunction in sepsis, we used the NET inhibitor (DNase1) and PAD4 inhibitor (Cl-amidine) to investigate the therapeutic effect of LG on septic rats and the effect on the coagulation function after NETs and PAD4 inhibition, respectively. Furthermore, DNase1 is a commonly used NET inhibitor, and studies have shown that DNase1 significantly inhibits NET formation in rats. Cl-amidine is a commonly used PAD4 inhibitor, and studies have shown that Cl-amidine inhibits PAD4 expression in rats [65]. The results showed that using NETs and PAD4 inhibitors had a therapeutic effect on septic rats and improved coagulation dysfunction in septic rats, consistent with previous reports that DNase1 and Cl-amidine have therapeutic effects on sepsis [66–68]. The therapeutic effects and the coagulation function of LG on septic rats are similar to those of NETs and PAD4 inhibitors alone. Furthermore, compared with the NETs or PAD4 inhibitor group alone, LG with NETs and PAD4 inhibitors show no more significant therapeutic effect.

Conclusively, our study confirmed that LG has therapeutic effects on septic rats; meanwhile, it also improves coagulation dysfunction in septic rats. Furthermore, the improvement of coagulation dysfunction in septic rats by LG was achieved through PAD4 inhibition, thus, inhibiting NET formation.

## Figures and Tables

**Table 1:** Changes in coagulation related indicators after LG treatment.

Groups	APTT (s)	PT (s)	TT (s)	FIB (*μ*g/L)
Sham	17.22 ± 5.02	14.98 ± 4.44	21.91 ± 3.25	3.52 ± 0.90
CLP	53.90 ± 15.6^##^	50.90 ± 20.37^##^	27.24 ± 3.69	2.10 ± 0.24^##^
LLG	35.94 ± 11.10	43.56 ± 18.05	25.64 ± 2.70	2.07 ± 0.51
MLG	32.33 ± 9.58^*∗∗*^	27.19 ± 12.98^*∗∗*^	25.12 ± 5.71	2.60 ± 0.61^*∗*^
HLG	28.36 ± 6.12^*∗∗*^	25.63 ± 7.18^*∗∗*^	22.51 ± 4.27^*∗*^	2.99 ± 0.76^*∗∗*^

The Sham, CLP, LLG, MLG, and HLG groups (*n* = 15, 8, 8, 10, and 11, respectively). Data are presented as the mean ± SD. ^##^*p* < 0.01 as compared to the Sham group; ^*∗*^*p* < 0.05 and ^*∗∗*^*p* < 0.01 as compared to the CLP group.

**Table 2:** Changes in coagulation related indicators after LG, DNase1, and Cl-amidine treatment.

Groups	*t*	PT (s)	TT (s)	FIB (*μ*g/L)
Sham	19.94 ± 4.09	20.30 ± 7.11	21.17 ± 4.46	3.09 ± 0.88
CLP	40.34 ± 12.48^##^	44.23 ± 7.94^##^	20.43 ± 5.12	1.76 ± 0.30^##^
LG	29.20 ± 5.90^*∗∗*^	30.42 ± 3.19^*∗∗*^	23.00 ± 8.57	2.86 ± 0.46^*∗∗*^
DNase1	29.70 ± 6.14^*∗*^	32.57 ± 6.47^*∗∗*^	23.91 ± 6.81	2.67 ± 0.47^*∗∗*^
LGD	29.29 ± 9.18^*∗∗*^	30.59 ± 2.03^*∗*^	24.08 ± 4.51	2.96 ± 0.70^*∗∗*^
Cl-amidine	23.98 ± 7.35^*∗*^	35.28 ± 9.34^*∗∗*^	25.46 ± 5.79	2.69 ± 0.51^*∗∗*^
LGCl	23.35 ± 5.36^*∗∗*^	34.33 ± 5.21^*∗∗*^	24.02 ± 8.63	2.55 ± 0.46^*∗∗*^

The Sham, CLP, LG, DNase1, LGDN, Cl-amidine, and LGCl groups (*n* = 15, 7, 10, 9, 10, 10, and 9, respectively).

## Data Availability

The datasets used and/or analyzed during the current study are available from the corresponding author upon reasonable request.

## Supplementary Materials

Supplementary Materials The reagents used in this study, the extraction protocol of neutrophils, and the information of antibodies for western blotting.
