# Supplementary material for: Liang-Ge Decoction Ameliorates Coagulation Dysfunction in Cecal Ligation and Puncture-Induced Sepsis Model Rats through Inhibiting PAD4-Dependent Neutrophil Extracellular Trap Formation
Source: Evid Based Complement Alternat Med. 2023 Apr 29;2023:5042953. doi: 10.1155/2023/5042953 (PMC10163969; doi:10.1155/2023/5042953)
Supplement: Supplementary Materials — The reagents used in this study, the extraction protocol of neutrophils, and the information of antibodies for western blotting. [file 5042953.f1.docx]

**Reagents**

Male SD rats (200 ± 20 g) were bought from Huafukang Bioscience (Beijing, China). DNase1 (cat.no.: D8071), rat blood neutrophil isolation kit (cat.no.: P9200) and DAPI (cat.no.:C0060) were purchased from Solarbio (Beijing, China). Cl-amidine (cat.no.: GC11032) was purchased from GlpBio Technology (USA). Enzyme-linked immunosorbent assay (ELISA) kits for rat interleukin (IL)-1β (EK301B), IL-6 (EK306), tumor necrosis factor alpha (TNF-α; EK382) were purchased from Multi Science Biotechnology (Hangzhou, China). ELISA kits for rat von Willebrand factor (vWF; cat.no.: ml003160), thrombomodulin (TM; cat.no.: ml059145), thrombin antithrombin complex (TAT; cat.no.: ml003154), platelet activating factor (PAF; cat.no.: ml003008), plasminogen activator inhibitor 1 (PAI-1; cat.no.: ml003024), and P-selectin (cat.no.: ml102808) were purchased from Enzyme-linked Biotechnology (Shanghai, China). Rabbit anti-thrombin (cat.no.: bs-1914R) was obtained from Biosynthesis Biotechnology (Beijing, China). Goat anti-rabbit IgG H&L(cat.no.: ab205718) and rabbit polyclonal to Histone H3 (cat.no.: ab5103)was purchased from Abcam (Shanghai, China). Myloperoxidase (MPO) mouse monoclonal (cat.no.: GB12224) was purchased from Servicebio (Wuhan, China). Dulbecco's modified Eagle medium (DMEM) culture media and Streptomycin/Penicillin were purchased from Gibco (Beijing, China). Fetal bovine serum (FBS) was obtained from Hyclone (Beijing, China).

**Neutrophil extraction**

4 mL of Reagent A was added to the collected anticoagulated blood, and then 2 mL of reagent C was carefully superimposed on Reagent A to form a gradient interface. The blood sample was laid flat on the liquid surface of the isolation solution, paying attention to keeping the interface between the two liquid surfaces clear. After centrifugation at room temperature for 20 min at 1000 g, two white cell circular and milky layers appeared, where the upper cell layer was the single nuclear cell, and the lower cell layer was the neutrophil layer. The neutrophil layer was carefully aspirated into a 15 mL clean centrifuge tube, where 10 mL PBS was added and centrifuged for 10 min at 250 g. After centrifugation, the precipitate was collected, and 5-mL PBS was added to resuspend the cells, centrifuged for 10 min at 250 g. The precipitate obtained was the neutrophils.

**Western blotting**

PAD4 polyclonal antibody (1:3000, 17373-1-AP, Proteintech)

β-actin monoclonal antibody (1:5000, 66009-1-Ig, Proteintech)

goat anti-rabbit IgG(H+L) (1: 10000, SA00001-2, Proteintech)

goat anti-mouse IgG(H+L) (1: 10000, SA00001-2, Proteintech)
